# Factors associated with the use of complementary and alternative therapies among patients with hypertension and type 2 diabetes mellitus in Western Jamaica: a cross-sectional study

**DOI:** 10.1186/s12906-020-03109-w

**Published:** 2020-10-17

**Authors:** Samuel Owusu, Yai-Ellen Gaye, Skyla Hall, Anna Junkins, Maira Sohail, Sarah Franklin, Maung Aung, Pauline E. Jolly

**Affiliations:** 1 Department of Epidemiology, School of Public Health, University of Alabama at Birmingham, 1665 University Boulevard, Birmingham, AL 35294-0022 USA; 2 Epidemiology Unit, Western Regional Health Authority, Ministry of Health, Montego Bay, St. James, Jamaica

**Keywords:** Complementary and alternative medicine, Hypertension, Type 2 diabetes, Herbal medicine, Jamaica

## Abstract

**Background:**

This study examined the prevalence and predictors of complementary and alternative medicine use among clinic patients with hypertension and/or type 2 diabetes mellitus in western Jamaica.

**Methods:**

A cross-sectional study using an investigator-administered questionnaire was conducted from May to August 2017. Data on sociodemographic factors, complementary and alternative medicine use, and knowledge and perceptions of complementary and alternative medicine were collected from the patients. Multivariable logistic regression analysis was used to examine associations between patient characteristics and knowledge and perceptions of complementary and alternative medicine and complementary and alternative medicine use.

**Results:**

A total of 362 patients were invited to participate and 345 (95.3%) completed the questionnaire; 311 (90.1%) had hypertension, 130 (37.7%) had type 2 diabetes mellitus and 96 (27.8%) had both diseases. Seventy-nine percent of the participants with hypertension and 65% with type 2 diabetes mellitus reported current use of complementary and alternative medicine. Self-reported knowledge of complementary and alternative medicine (none/poor vs average/good/excellent) was significantly associated with complementary and alternative medicine use for hypertension (AOR = 0.33, 95% CI = 0.13–0.87) and type 2 diabetes mellitus (AOR = 0.06, 95% CI = 0.01–0.37). Believing that complementary and alternative medicine is a natural method for treating hypertension was significantly associated with complementary and alternative medicine use among patients with hypertension (AOR = 3.9, 95% CI = 1.26–12.00), and belief that it is acceptable to use prescription medication and complementary and alternative medicine simultaneously was significantly associated with complementary and alternative medicine use among patients with type 2 diabetes mellitus (AOR =7.19, CI = 1.34–38.52).

**Conclusions:**

Participants’ perceptions of their knowledge and beliefs regarding complementary and alternative medicine strongly influence their use of complementary and alternative medicine. These findings can be used in designing educational interventions to promote the proper use, and mitigate detrimental effects, of complementary and alternative medicine in this population.

## Background

Hypertension (HTN) and Type 2 Diabetes Mellitus (T2DM) are major health problems globally. HTN is one of the most common non-communicable diseases, affecting up to 20% of the world’s adult population [1]. It was estimated that in 2017, 424 million people worldwide were suffering from Diabetes Mellitus (DM), with 50% undiagnosed and believed to be unaware of their illness [2]. The prevalence of DM is projected to rise to 628 million by 2045 [2]. T2DM accounts for 90% of cases of DM, and chronically affected patients can suffer from retinopathy, nephropathy, neuropathy, HTN, dyslipidemia, myocardial infarction, and stroke [3].

Several types of complementary and alternative medicines (CAM) are used worldwide for the treatment of HTN and T2DM. CAM is defined as “practices, approaches, knowledge, and beliefs that incorporate plant, animal and mineral-based medicines, spiritual therapies, manual techniques, and exercise” [4]. Individuals may choose an alternative or “natural” medical treatment because of beliefs or negative experiences with the mainstream medical community, lack of success with prescribed medicine, influence from friends and family, or negative experiences with healthcare providers (HCPs) [5]. In addition, CAM may be more easily accessible and less expensive than prescription medications [5]. Previous research shows that up to 80% of people living with T2DM use CAM. CAM use varied from 24% in the United Kingdom to 80% in Africa [6]. Most diabetic patients commonly use herbal medicine, nutritional supplements, diet modifications, spiritual healing, and relaxation techniques [4]. Despite the widespread use, a high proportion of patients might not disclose CAM use to their HCPs [7].

HTN has become a growing major public health problem for Caribbean countries in this century, with prevalence rates ranging from 26% for those over 25 years, and up to 55% for those over 40 years [8]. Half of the population aged ≥60 years is estimated to be living with HTN in Latin America and the Caribbean. People of black racial origin are at higher risk for developing HTN; therefore, Caribbean countries with predominately black populations, such as Jamaica and Barbados have higher prevalence rates of HTN [9]. In Jamaica, the estimated prevalence rate of HTN for persons aged 15–74 years is 25% [9]. Females were shown to be three times more likely to develop HTN than males and obese individuals were four times more likely to develop HTN than those with normal weight [10]. Other risk factors for HTN include: increasing age, family history of HTN, nutritional intake, salt intake, chronic stress, physical activity, and T2DM [9, 10].

T2DM has become an increasing problem in Latin America and the Caribbean in recent years, with certain Latin American countries having some of the highest prevalence rates globally [11]. In 2017, the total number of diagnosed cases of T2DM among adults 20–79 years in Jamaica was 209,300 [12]. The rate of T2DM cases increases with age. In 2015, Jamaicans aged 55–79-years had prevalence rates of T2DM ranging between 20 and 30%; those aged 20–34 years had prevalence rates of only 3–7% [12]. The cost of DM-related health care for adults in Latin America and Caribbean countries in 2000 was estimated at 65 billion United States Dollars (USD) [13]. Modifications in lifestyle such as exercise, weight loss, proper nutrition, and a low-fat diet are important in the management of T2DM.

“Bush” or herbal medicine use in the Caribbean is well documented. Herbs are widely used in treating conditions ranging from the common cold to chronic conditions such as HTN and T2DM [14–16]. Jamaican history shows that there has been a very high reliance on using medicinal plants as treatments for ailments [17]. A study conducted among black Caribbean patients found that 72.6% of participants reported regular use of herbal medicine to treat an illness or maintain good health in the past 12 months [15]. Of those who reported herbal medicine use, 77% reported using herbal medicine to treat respiratory illnesses and 53% to treat gastrointestinal problems [15]. Smaller percentages of participants reported the use of herbal treatments for HTN (15%) and T2DM (4%) [15]. Another study that examined the use of bush medicine in Trinidad and Tobago found that 42% of participants reported using herbs, and 24% reported using herbs for T2DM [16]. A study conducted in Jamaica in 2006 on the concomitant use of prescription medicines and herbs for a variety of ailments found that 83.2 and 79.5% of participants practiced concomitant herb-prescription medicine use for DM and HTN, respectively [18].

However, recent scientific literature on the use of CAM in the treatment of HTN and T2DM from the Caribbean, and Jamaica in particular, is sparse. Therefore, we conducted this research to investigate CAM use among patients diagnosed with HTN and T2DM in western Jamaica. We investigated associations between knowledge and perceptions of CAM and CAM use among the patients. The findings from this study can be used in the development of educational interventions that promote the appropriate use, and mitigate detrimental effects, of CAM in treating HTN and T2DM.

## Methods

### Study design, setting, and population

A cross-sectional study was conducted among patients ≥18 years of age attending any of seven chronic disease clinics for HTN and/or T2DM in the four parishes (St. James, Westmoreland, Hanover and Trelawny) under the Western Regional Health Authority (WRHA) of Jamaica. Nurses in these clinics informed eligible patients about the study, and those who expressed willingness to participate were directed to the research staff who provided further information and obtained informed consent from all patients who volunteered to participate.

### Inclusion and exclusion criteria

To be eligible to participate in the study patients had to be adults, ≥18 years of age who had been diagnosed with HTN and/or T2DM and were attending a chronic disease clinic in one of the four parishes under the WRHA. Patients who did not meet these inclusion criteria were excluded. A signed informed consent was obtained from each patient who agreed to participate.

### Sample size and sampling method

Based on previous estimates of concomitant use of herb and prescription medication among hypertensive (79.5%) and diabetic (83.2%) patients in Jamaica, the sample size for the current study was determined to be a minimum of 251 hypertensive and 215 diabetic patients with a 95% confidence limit and 5% margin of error [18]. The sample was calculated using the online calculator EpiTools by Ausvet© (http://epitools.ausvet.com.au). A convenience sampling method was used to recruit patients at the clinics during the study period. Patients attending the chronic disease clinics who met the inclusion criteria were eligible to participate and were included in the study if they agreed and signed the informed consent.

### Questionnaire development and data collection

An investigator-administered questionnaire was used to collect data on sociodemographic factors (age, education, employment status, income, and residence), CAM use, knowledge and perception of CAM, and concomitant use of prescription medicine and CAM. Questions on education level, income, and occupation were important since previous studies found that income and education level can influence knowledge and use of CAM. Questions on knowledge of CAM, perception of CAM, and concomitant use of CAM with prescription medicines were adapted from questions used in published studies on CAM that were conducted in Jamaica, as well as other Caribbean and non-Caribbean countries. Questions were included to allow participants to list the types of CAM they were using for HTN or T2DM. The questionnaire was reviewed by Jamaican HCPs and revised. It was then pilot tested among 10 clinic patients in Jamaica similar to the ones recruited for the study and revised before use.

### Data analysis

Bivariate analyses using chi-square tests were conducted to determine statistically significant differences between sociodemographic characteristics, prescription medication use, self-reported knowledge of CAM, and perception of CAM with CAM use among hypertensive and diabetic patients separately. Sociodemographic variables included in the analyses were age, sex, parish, education, religious affiliation, marital status, occupation, monthly income, number of adults living in a household (NALH), number of children living in a household (NCLH), and time since HTN or T2DM diagnosis.

Logistic regression analysis estimated odds ratios (OR), adjusted odds ratios (AOR), and corresponding 95% confidence intervals predicting CAM use for treatment of HTN and T2DM separately. Multivariable models included variables that were statistically significant in bivariate analyses. Statistical Analysis System (SAS, Cary, North Carolina, USA) version 9.4 was used for the analyses.

### Ethics approval

The study was reviewed and approved by the Institutional Review Board at the University of Alabama at Birmingham and the Western Regional Health Authority in Jamaica.

## Results

### Sociodemographic characteristics associated with CAM use for HTN and T2DM (Table 1)

Among the 345 patients included in the study, 311 had HTN, 130 had T2DM, and 96 had both diseases; about half (52%) were ≥ 60 years of age and 76.5% were female (Table 1). Current use of CAM was reported by 79.4% of participants with HTN and by 65.4% with T2DM (Table 1). Sex and parental status were marginally associated with CAM use for HTN (Table 1). A greater proportion of CAM users compared to non-CAM users were female (80% vs. 69%). Among patients with T2DM, age and the number of adults in the household were marginally associated with use of CAM; 71% of those who did not use CAM were ≥ 60 years of age.

**Table 1:** Sociodemographic characteristics of patients with hypertension (HTN) and Type 2 diabetes mellitus (T2DM) by CAM use

Variables	All (*n* = 345)	HTN (*n* = 311)			T2DM (*n* = 130)		
		Used CAM 247 (79.4)	Did not use CAM 64 (20.6)	*p*-value†	Used CAM 85 (65.4)	Did not use CAM 45 (34.6)	*p*-value†
Age (years)				0.116			**0.052**
18–49	85 (24.9)	64 (26.1)	9 (14.3)		17 (20.2)	7 (15.6)	
50–59	80 (23.5)	55 (22.5)	14 (22.2)		25 (29.8)	6 (13.3)	
≥ 60	176 (51.6)	126 (51.4)	40 (63.5)		42 (50.0)	32 (71.1)	
Sex				**0.063**			0.526
Male	81 (23.5)	50 (20.3)	20 (31.3)		22 (25.9)	14 (31.1)	
Female	263 (76.5)	196 (79.7)	44 (68.8)		63 (74.1)	31 (68.9)	
Parish				0.678			0.626
Hanover	21 (6.1)	14 (5.7)	5 (7.8)		3 (3.5)	1 (2.2)	
St. James	188 (54.5)	140 (56.7)	32 (50.0)		39 (45.9)	25 (55.6)	
Westmoreland	109 (31.6)	74 (30.0)	23 (35.9)		35 (41.2)	17 (37.8)	
Trelawny	27 (7.8)	19 (7.7)	4 (6.3)		8 (9.4)	2 (4.4)	
Marital status				0.895			0.199
Never married	152 (44.0)	105 (42.5)	26 (40.6)		43 (50.6)	18 (40.0)	
Married/common law	132 (38.3)	98 (39.7)	25 (39.1)		28 (32.9)	22 (48.9)	
Divorced/Widowed	61 (17.7)	44 (17.8)	13 (20.3)		14 (16.5)	5 (11.1)	
Education				0.763			0.842
Primary or less	165 (48.0)	121 (49.0)	30 (46.9)		39 (45.9)	21 (47.7)	
Some or completed Secondary/College/University	179 (52.0)	126 (51.0)	34 (53.1)		46 (54.1)	23 (52.3)	
Religious affiliation				0.663			0.427
Catholic/Protestant Christian	235 (68.1)	167 (67.6)	41 (64.1)		64 (75.3)	29 (64.4)	
Adventist	72 (20.9)	55 (22.3)	14 (21.9)		13 (15.3)	10 (22.2)	
Muslim/Jehovah Witness/	38 (11.0)	25 (10.1)	9 (14.1)		8 (9.4)	6 (13.3)	
Occupation				0.458			0.762
Unskilled	269 (78.0)	190 (76.9)	52 (81.3)		70 (82.4)	38 (84.4)	
Professional/Skilled/ Clerical/Other	76 (22.0)	57 (23.1)	12 (18.8)		15 (17.7)	7 (15.6)	
Income				0.454			0.347
No Income	190 (55.1)	130 (52.6)	39 (60.9)		53 (62.4)	33 (73.3)	
< J$24,800	94 (27.3)	71 (28.7)	14 (21.9)		20 (23.5)	9 (20.0)	
≥ J$24,801	61 (17.7)	46 (18.6)	11 (17.2)		12 (14.1)	3 (6.7)	
Have children	319 (92.7)	226 (91.9)	63 (98.4)	**0.063**	75 (88.2)	42 (93.3)	0.357
Number of adults in household							**0.068**
1	79 (23.1)	51 (20.8)	16 (25.4)	0.561	27 (31.8)	15 (34.1)	
2–3	172 (50.3)	125 (51.0)	33 (52.4)		36 (42.4)	25 (56.8)	
≥ 4	91 (26.6)	69 (28.2)	14 (22.2)		22 (25.9)	4 (9.1)	
Number of children in household				0.318			0.801
≤ 1	263 (77.1)	183 (75.3)	52 (81.3)		72 (84.7)	38 (86.4)	
≥ 2	78 (22.9)	60 (24.7)	12 (18.8)		13 (15.3)	6 (13.6)	
Time of diagnosis				0.358			0.259
< 10 years ago	150 (48.9)	122 (50.2)	28 (43.8)		38 (45.2)	24 (55.8)	
≥ 10 years ago	157 (51.1)	121 (49.8)	36 (56.2)		46 (54.8)	19 (44.2)	

† *P*-value based on chi-square

-$24,800 = Jamaican minimum wage at the time of the study, *CAM* Complementary and alternative medicine

### Most common alternative therapies used for treatment of HTN and T2DM

The most common CAM method used (answers are not mutually exclusive) by participants with HTN was herbal medicine (72.1%); this was followed by diet modification (46.6%), exercise (40.1%), nutritional supplements (15.4%), relaxation techniques (9.3%), spiritual healing (6.5%) and manual techniques (0.8%). For T2DM, the most common CAM method reported was herbal medicine (65.9%); this was followed by diet modification (52.9%), exercise (41.2%), relaxation techniques and spiritual healing (10.6% each), and nutritional supplements (9.4%). The most commonly reported herbal medicines used for HTN were, Garlic (*Allium sativum)*, Soursop (*Annona muricate)*, Cerasee (*Momordica charantia)*, Guinea Hen Weed (*Petiveria alliacea),* and Coconut water (*Cocos nucifera)* with lime (*Citrus aurantiifolia).* For T2DM, the most common herbal medicines reported were Cerasee (*Momordica charantia)*, Bird Pepper (*Capsicum annuum)*, Coconut water (*Cocos nucifera)*, Pound-cake Bush (*Parthenium hysterophorus),* and King of the Forest (*Cassia alata)*.

### Prescription medication use associated with CAM use for HTN and T2DM (Table 2)

The majority of patients in the study (94%) were prescribed medication for HTN (91.3%) or DM (93.8%), and approximately 58% received their prescription medication from the hospital or health center (~ 58%); approximately 76–77% reported filling their HTN prescription on time (Table 2). Among participants with HTN, those who did not use CAM were more likely to report that their prescription medication was controlling their HTN and were less likely to discontinue medication when their blood pressure was normal compared to CAM users. Among participants with T2DM, those who used CAM were more likely to pay for their medication (76.3% vs. 53.9%; *p* = 0.014) and much more likely to report that they experience side effects from their prescription medication versus those who did not use CAM (41.0% vs. 18.0%; *p* = 0.013).

**Table 2:** Prescription medication association with CAM use among patients with hypertension (HTN) and type 2 diabetes mellitus (T2DM)

Variables	Hypertension (*n* = 311)			Type 2 Diabetes Mellitus (*n* = 130)		
	Used CAM 247 (79.4)	Did not use CAM 64 (20.6)	*p*-value†	Used CAM 85 (65.3)	Did not use CAM 45 (34.6)	*p*-value
Prescribed medicine for HTN/DM			**0.080**			0.860
Yes	221 (89.8)	61 (96.8)		80 (94.1)	42 (93.3)	
No	25 (10.2)	2 (3.2)		5 (5.9)	3 (6.7)	
Provider of prescription medicine			0.936			0.170
Hospital/Health center	129 (58.6)	36 (58.1)		44 (58.7)	30 (71.4)	
Pharmacy	91 (41.4)	26 (41.9)		31 (41.3)	12 (28.6)	
Pay for medication			0.220			**0.014**
No	71 (32.0)	25 (40.3)		18 (23.7)	18 (46.2)	
Yes	151 (68.0)	37 (59.7)		58 (76.3)	21 (53.9)	
Refill medicine on-time			0.787			0.163
Yes	172 (75.8)	48 (77.4)		57 (73.1)	33 (84.6)	
No/Sometimes	55 (24.2)	14 (22.6)		21 (26.9)	6 (15.4)	
Take medicine as prescribed			0.299			0.153
Always	110 (48.3)	34 (55.7)		41 (51.3)	26 (65.0)	
Not Always	118 (51.8)	27 (44.3)		39 (48.8)	14 (35.0)	
Medicine controlling disease			**0.051**			0.937
Yes	172 (77.5)	55 (88.7)		67 (85.9)	35 (85.4)	
No/Sometimes	50 (22.5)	7 (11.3)		11 (14.1)	6 (14.6)	
Discontinues medicine when BP normal			**0.099**			
Yes/Sometimes	74 (33.2)	13 (22.0)				
No	149 (66.8)	46 (78.0)				
Experience side effects from medicine			0.781			**0.013**
Yes	73 (33.0)	19 (31.2)		32 (41.0)	7 (18.0)	
No	148 (67.0)	42 (68.9)		46 (59.0)	32 (82.1)	

*CAM* Complementary and alternative medicine

### Self-reported knowledge, perceptions, and use of CAM for HTN and T2DM (Table 3)

Most participants reported receiving information about CAM from friends, family members, or the media (Table 3). Several factors related to knowledge and perceptions of CAM were found to be associated with CAM use by both participants with HTN and T2DM. These include: 1) self-reported knowledge of CAM, 2) previously discussing CAM with a HCP, 3) acceptability of simultaneous use of CAM and prescription medicine, and 4) acceptability of discontinuing use of prescription medication without consulting a HCP if side effects are experienced (Table 3). Additionally, the source of CAM information, belief that CAM is a natural method for treating HTN, and a perception that CAM should always/sometimes be used instead of prescription medicine were associated with CAM use among patients with HTN. For patients with T2DM, having received information on CAM and a perception that CAM is more effective than prescription medication at treating DM were associated with CAM use (Table 3).

**Table 3:** Self-reported knowledge and perceptions of CAM and CAM use for hypertension (HTN) and type 2 diabetes mellitus (T2DM)

	HTN (*n* = 311)			T2DM (*n* = 130)		
	Used CAM 247 (79.4)	Did not use CAM 64 (20.6)	*p*-value†	Used CAM 85 (65.3)	Did not use CAM 45 (34.6)	*p*-value
Received information about CAM	51 (79.7)	14 (63.6)	0.131	66 (86.8)	25 (27.5)	**0.002**
Source of information on CAM			**0.076**			0.834
HCP/clinic staff	38 (21.0)	3 (8.3)		10 (21.3)	4 (19.1)	
Friend/family member/Media	143 (79.0)	33 (91.7)		37 (78.7)	17 (81.0)	
Heard positive or negative information about CAM^a^			0.271			0.252
Yes	210 (90.9)	42 (85.7)		74 (93.7)	35 (87.5)	
No	21 (9.1)	7 (14.3)		5 (6.3)	5 (12.5)	
Self-reported knowledge of CAM			**< 0.0001**			**< 0.001**
None/Some	119 (49.2)	48 (81.4)		37 (45.1)	35 (83.3)	
Average/Good/Excellent	123 (50.8)	11 (18.6)		45 (54.9)	7 (16.7)	
Discussed CAM with healthcare provider			**0.004**			**0.008**
Yes	62 (25.9)	5 (8.3)		21 (28.4)	3 (7.3)	
No	177 (74.1)	55 (91.7)		53 (71.6)	38 (92.7)	
Always discuss any CAM for your condition with your HCP			0.211			0.356
Yes	170 (70.5)	37 (59.7)		55 (67.9)	25 (59.5)	
No	70 (29.1)	25 (40.3)		26 (32.1)	17 (40.5)	
Perceives that a normal blood pressure reading means that.			0.941			
Cured of disease	11 (5.0)	3 (5.3)				
Blood pressure is normal at the time, but disease still present	208 (95.0)	54 (94.7)				
Believes CAM is a natural method for treating the disease	165 (68.5)	30 (48.4)	**0.010**	60 (73.2)	27 (62.8)	0.231
Perceives that it is acceptable to use prescription medication and CAM simultaneously			**0.058**			**0.083**
Yes	72 (29.8)	11 (16.7)		27 (33.3)	8 (18.6)	
No	170 (70.3)	51 (83.3)		54 (66.7)	35 (81.4)	
Perceives that it is acceptable to stop taking medicine without consulting HCP if experiencing unpleasant side effects			**0.018**			**0.031**
Yes	80 (33.2)	11 (16.7)		30 (37.5)	8 (18.6)	
No	161 (66.8)	51 (83.3)		50 (62.5)	35 (81.4)	
Perceives that CAM should always be used instead of prescription medication			**0.003**			0.462
Yes	101 (41.6)	13 (21.0)		27 (32.9)	17 (39.5)	
No	142 (58.4)	49 (79.0)		55 (67.1)	26 (60.5)	
Perceives that CAM is more effective at treating disease than prescription			0.105			**0.062**
Yes	89 (36.8)	16 (25.8)		30 (36.6)	14 (32.6)	
No	153 (63.2)	46 (74.2)		44 (53.7)	18 (41.9)	

^a^Including that CAM is as good as or better than prescription medication, is effective, accessible, does not have side effects, and is inexpensive, is worse than prescription medication, is ineffective, has side effects, and is expensive; *CAM* Complementary and alternative medicine

### Predicted odds of using CAM for HTN (Table 4)

Among participants with HTN, self-reported knowledge of CAM and believing that CAM is a natural method for treating HTN were significantly associated with use of CAM, even after adjustment for covariates (Table 4). Participants who reported none/some knowledge of CAM were 67% less likely to use CAM compared to those who reported average/good/excellent knowledge of CAM (AOR = 0.33, CI = 0.13–0.87). Those who believed that CAM is a natural method for treating HTN were 3.9 times more likely to use CAM (AOR = 3.9, CI = 1.26–12.00).

**Table 4:** Odds ratios (OR) and 95% confidence intervals (CI) for the associations of knowledge and perception of CAM and CAM use for the treatment of hypertension (HTN)^a^

	Crude OR (95% CI)	Adjusted^b^ OR (95% CI)
Sex		
Male	0.56 (0.30–1.04)	0.51 (0.16–1.57)
Female	Ref	Ref
Children		
Yes	0.18 (0.02–1.36)	QCS^c^
No	Ref	Ref
Prescribed medicine		
Yes	0.29 (0.07–1.26)	QCS^c^
No	Ref	Ref
Medicine controlling disease		
Yes	0.44 (0.19–1.02)	0.61 (0.19–1.95)
No	Ref	Ref
Discontinues medicine when BP normal		
Yes	1.76 (0.89–3.45)	1.13 (0.40–3.15)
No	Ref	Ref
Received information about CAM from …		
HCP/clinic staff	2.92 (0.85–10.05)	1.81 (0.34–9.61)
Friend/family member/Media	Ref	Ref
Self-reported of knowledge of CAM		
None/Some	**0.22 (0.11–0.45)**	**0.33 (0.13–0.87)**
Average/Good/Excellent	Ref	Ref
Discussed CAM with HCP		
Yes	**3.85 (1.48–10.06)**	2.85 (0.68–12.00)
No	Ref	Ref
Believes CAM is a natural method for treating disease		
Yes	**2.35 (1.33–4.14)**	**3.90 (1.26–12.00)**
No	Ref	Ref
Perceives that it is acceptable to use prescription medication and CAM simultaneously		
Yes	1.96 (0.97–3.98)	1.60 (0.55–4.70)
No	Ref	Ref
Perceives that it is acceptable to stop taking prescription medication without consulting HCP if experiencing unpleasant side effects		
Yes	**2.30 (1.14–4.66)**	1.10 (0.39–3.09)
No	Ref	Ref
Perceives that CAM should always be used instead of prescription medication		
Yes	**2.68 (1.38–5.20)**	1.21 (0.55–2.68)
No	Ref	Ref

^a^ Estimated using logistic regression

^b^ Adjusted model includes significant variables in bivariate analyses: sex, parental status, prescription hypertensive medicine, medicine controlling hypertension, stop taking medicine if BP normal, perceives that it is acceptable to discontinue prescription medicine without consulting HCP, source from which have received information about CAM, previously discussing CAM with HCP, self-reported knowledge of CAM, perceives that CAM is a natural treatment method, perceives that it is acceptable to use prescription medication and CAM simultaneously to treat hypertension, and perceives that CAM should be used instead of prescription medication all or some of the time

^c^Quasi-complete separation (QCS), *CAM* Complementary and alternative medicine

### Predicted odds of using CAM for T2DM (Table 5)

Among participants with T2DM, self-reported knowledge of CAM and acceptability of simultaneous use of prescription medicine and CAM were associated with CAM use (Table 5). T2DM patients who reported none/some knowledge of CAM were 94% less likely to use CAM compared to those who reported average/good/excellent knowledge of CAM (AOR = 0.06, CI = 0.01–0.37). Those who perceived that it is acceptable to use prescription medication and CAM simultaneously were 7.19 times more likely to use CAM compared to those who did not (AOR = 7.9, CI = 1.34–38.52).

**Table 5:** Odds ratios (OR) and 95% confidence intervals (CI) for the associations of knowledge and perception of CAM and CAM use for the treatment of type 2 diabetes mellitus (T2DM) ^a^

	Crude OR (95% CI)	Adjusted^b^ OR (95% CI)
Age		
18–49	1.77 (0.66–4.76)	3.51 (0.66–18.81)
50–59	**2.60 (1.00–6.74)**	3.97 (0.87–18.05)
≥ 60	Ref	Ref
Number of adults in household		
0 to 1	0.33 (0.10–1.13)	2.92 (0.45–19.17)
2 to 3	**0.27 (0.08–0.86)**	0.75 (0.13–4.55)
≥ 4	Ref	Ref
Medication cost		
No cost/free	**0.36 (0.16–0.82)**	0.34 (0.09–1.22)
Have to pay	Ref	Ref
Side effects from medicine		
Yes	**3.21 (1.27–8.14)**	1.65 (0.43–6.39)
No	Ref	Ref
Received information about CAM		
Yes	**3.96 (1.57–9.97)**	1.61 (0.37–7.03)
No	Ref	Ref
Self-reported of knowledge of CAM		
None/Some	**0.16 (0.07–0.41)**	**0.06 (0.01–0.37)**
Average/Good/Excellent	Ref	Ref
Discussed CAM with HCP		
Yes	**4.93 (1.37–17.70)**	4.71 (0.75–29.46)
No	Ref	Ref
Perceives that it is acceptable to use prescription medication and CAM simultaneously		
Yes	2.15 (0.88–5.26)	**7.19 (1.34–38.52)**
No	Ref	Ref
Perceives that it is acceptable to stop taking medicine without consulting HCP if experiencing unpleasant side effects		
Yes	**2.71 (1.12–6.60)**	0.89 (0.20–3.91)
No	Ref	Ref
Perceives that CAM is more effective at treating disease than prescription medication		
Yes	1.17 (0.54–2.56)	0.99 (0.40–2.45)
No	Ref	Ref

^a^ Estimated using logistic regression

^b^ Adjusted model includes significant variables in bivariate analyses: age, number of adults in household, cost of medication, side effects from medicine, perceives that it is acceptable to discontinue prescription medicine without consulting HCP, previously receiving information about CAM, previously discussing CAM with their HCP, self-reported knowledge of CAM, perceiving that it is acceptable to use prescription medication and CAM simultaneously, and perceives that CAM is more effective at treating disease than prescription;

*CAM* Complementary and alternative medicine

## Discussion

This study describes the prevalence and factors associated with CAM use by patients for treatment of HTN and T2DM in western Jamaica. More than one-quarter (28%) of the patients had both HTN and T2DM. HTN and T2DM frequently coexist, leading to increase in the risk of life-threatening complications. The high proportion of females (76.5%) in the study can be explained by the fact that females are a high-risk group for developing HTN in Jamaica. Previous research in Jamaica suggests that females are three times more likely to develop HTN compared to males [10]. Thus, our enrollment of 76.5% females exactly matches this reported prevalence.

Our finding of a high prevalence of CAM use among patients with both HTN and T2DM is consistent with that of a study that reported herbal medicine to treat ailments and maintain health by 72.6% of Jamaicans [15]. Our results are also consistent with the findings of some studies conducted globally that show CAM usage of 17–80%, with the highest rates in Africa [6]. The reason for the high use of herbal medicine in Jamaica could be attributed to the fact that herbs are a part of the daily lives of many Jamaicans since they are often consumed as tea [19]. However, the increased interest, belief, and use of CAM in Jamaica in the mid-late 1990s, and Jamaica’s poor economic situation, may have helped to facilitate the increased use of herbs [20, 21]. The medications associated with the treatment for HTN and T2DM are lengthy and costly. The study by Delgoda et al., found that those of high socioeconomic status and those with health insurance had much lower use of combined herb and prescription medication, further strengthening the idea that the boom in herbal use was in part due to financial reasons [18].

In this study, over 60% of participants with T2DM and 70% with HTN used CAM simultaneously with prescription medicine. A previous study found that almost 80% of patients with HTN and 83% with T2DM practiced concomitant use of CAM and prescription drugs; 94% of respondents believed that there was no harm in taking CAM and prescription medication concomitantly [18]. Over two-thirds of participants in our study said that it was unacceptable to use prescription medicine and CAM simultaneously. However, patients with T2DM who believed that simultaneous use of CAM and prescription medicine was acceptable were seven times more likely to use CAM, and patients with HTN who believed that CAM is a natural method for treating disease were almost 4 times more likely to use CAM. These findings clearly show that patients’ beliefs regarding CAM strongly determine their use CAM and point to the need for appropriate counseling on the use of CAM, especially along with prescription medication, in order to mitigate possible harmful effects of simultaneous use of CAM and prescription medicine. For example, garlic (*Allium sativum*) which is commonly used in Jamaica to treat HTN and T2DM, has been shown to have antihypertensive, hypoglycemic, and lipid-lowering properties and therefore could cause adverse health effects if taken simultaneously with prescribed medication for HTN and T2DM [22, 23].

In the current study, self-reported knowledge of CAM was significantly associated with CAM use among patients with HTN and T2DM. In both groups, patients who reported that they had no/poor knowledge of CAM were significantly less likely to use CAM than those who reported average/good/excellent knowledge of CAM. Thus, use of CAM by patients seems to be driven by confidence regarding knowledge of CAM. Future studies should assess self-reported knowledge compared with tested knowledge of CAM among participants. This would allow identification of misconceptions regarding CAM and for educational interventions that provide accurate information on CAM to be developed and conducted. In addition to educational interventions on CAM, increasing resources within communities for easy access to routine blood pressure checks and blood glucose readings will help patients to monitor their blood pressure and blood glucose levels frequently. This could help them to understand their illness and document the effects of prescription medicine in controlling their HTN and/or T2DM.

The gap in patient-HCP dialogue regarding CAM use is of particular concern. Only about 20% of our participants reported that they discussed CAM use with their HCPs. Other studies report that only 18–19% of medical practitioners are aware of herbal use by their patients [8, 24]. This gap in communication between patients and HCPs may be attributed to the fact that many patients expect negative reactions from HCPs about their use of CAM and prefer not to disclose such information [15]. On the other hand, this lack of communication could occur because HCPs do not ask patients about their CAM use [2]. The Jamaican study by Delgoda et al., reported that only 11.3% of participants who used herbs were asked about CAM use by their doctors. Our results indicate that among the participants who used CAM, only 19–20% reported that they had received information about CAM from their HCP.

In accordance with the World Health Organization (WHO) 2008 Beijing Declaration, which promotes the safe and effective use of herbal remedies, there is a need for more publicly available information regarding the safe use of herbal remedies [24]. In order to promote appropriate knowledge of CAM in Jamaica, there is need for collaboration between the Ministry of Health and health educational institutions (schools of nursing, medicine, nutrition, pharmacy and public health) on training in CAM and communication with patients.

### Limitations

There are limitations that should be considered in interpreting the results of this study. First, the sample of patients with T2DM is small and may affect generalizability of the results from the current study to the population of diabetic patients in the Western region or other regions of Jamaica. Secondly, the study sample represents only patients who attended the chronic disease clinics in the WRHA that were included in the study; therefore, the results may not be generalizable to patients attending other clinics or parishes that were not sampled. Additionally, the data were self-reported and as a result might be subject to social desirability bias.

## Conclusions

Regardless of the limitations, this study identified factors such as self-reported knowledge of CAM, belief that CAM is a natural method for treating HTN, and belief that it is acceptable to use CAM and prescription medication simultaneously to treat T2DM, to be significantly associated with CAM use. These findings strongly suggest the need for educational interventions for both patients and HCPs to address the gaps in knowledge and communication of CAM and to ensure proper use of CAM in treating HTN and T2DM. This is important for the appropriate management of these major debilitating diseases in the Jamaican society.

## Data Availability

The datasets generated and/or analyzed during the current study are not publicly available due to the fact that further analysis is still being conducted on the data but are available from the corresponding author on reasonable request.

## Abbreviations

HTN: Hypertension
T2DM: Type 2 Diabetes Mellitus
DM: Diabetes Mellitus
CAM: Complementary and alternative medicine
HCP: Healthcare provider
USD: United States Dollar.
WRHA: Western Regional Health Authority
NALH: Number of adults living in household
NCLH: Number of children living in household
OR: Odds ratio
AOR: Adjusted odds ratio
95% CI: 95% Confidence interval
